# Deep-diving pilot whales make cheap, but powerful, echolocation clicks with 50 µL of air

**DOI:** 10.1038/s41598-019-51619-6

**Published:** 2019-10-31

**Authors:** Ilias Foskolos, Natacha Aguilar de Soto, Peter Teglberg Madsen, Mark Johnson

**Affiliations:** 1 Sea Mammal Research Unit, University of St Andrews, St Andrews, Fife KY16 8LB United Kingdom; 2 Dept. Biology, University of La Laguna, Tenerife, Spain; 3 Department of Bioscience, Aarhus University, Aarhus, Denmark; 4 Aarhus Institute for Advanced Studies, Aarhus University, Denmark

**Keywords:** Ecophysiology, Animal physiology

## Abstract

Echolocating toothed whales produce powerful clicks pneumatically to detect prey in the deep sea where this long-range sensory channel makes them formidable top predators. However, air supplies for sound production compress with depth following Boyle’s law suggesting that deep-diving whales must use very small air volumes per echolocation click to facilitate continuous sensory flow in foraging dives. Here we test this hypothesis by analysing click-induced acoustic resonances in the nasal air sacs, recorded by biologging tags. Using 27000 clicks from 102 dives of 23 tagged pilot whales (*Globicephala macrorhynchus)*, we show that click production requires only 50 µL of air/click at 500 m depth increasing gradually to 100 µL at 1000 m. With such small air volumes, the metabolic cost of sound production is on the order of 40 J per dive which is a negligible fraction of the field metabolic rate. Nonetheless, whales must make frequent pauses in echolocation to recycle air between nasal sacs. Thus, frugal use of air and periodic recycling of very limited air volumes enable pilot whales, and likely other toothed whales, to echolocate cheaply and almost continuously throughout foraging dives, providing them with a strong sensory advantage in diverse aquatic habitats.

## Introduction

The evolution of echolocation has enabled toothed whales to access vast food resources in the deep ocean where this high resolution active sensory system gives a critical advantage over energy- and light-limited prey^1^. Echolocation requires the production of 1000’s of powerful sound transients per hour to insonify prey in deep breath-hold dives. But despite spending most of their lives submerged, toothed whales use air to produce these sounds. Echolocation clicks are produced by passing compressed air from the nasopharyngeal air space through the bony nares to the vestibular sacs via the phonic lips^2^ where highly innervated facial and nasal muscles control air flow^3^ (Fig. S1). The passage of air across the phonic lips produces a short click which is then collimated and filtered by the skull, air sacs and melon, before radiating into the water in a narrow high intensity beam^4–6^ (Fig. S1). Clicks produced by deep-divers such as sperm whales *Physeter macrocephalus* are the most intense sounds ever recorded from animals^7^ and enable long-range detection of prey in an extensive three dimensional habitat^8^. However, in spite of the ecological importance of echolocation for toothed whales, little is known about the energetic costs of this sensory system or the behavioural and biomechanical constraints imposed by the need for continuous pneumatic sound production in deep dives^9,10^.

The use of air as the motive force for underwater sound production, inherited from terrestrial ancestors, imposes profound constraints on deep-diving toothed whales. Air taken from the surface is compressed by hydrostatic pressure according to Boyle’s law so that at 1000 m only 1% of the surface air volume remains. Thus, although significant energy must be expended to dive away from the surface against the buoyancy force of air in the lungs^11^, this effort yields only a small supply of air at foraging depths of deep-diving toothed whales^9^. This compressed gas is not a useful source of oxygen to fuel aerobic dives^12^ and may, if managed poorly, give rise to decompression sickness^13,14^. Accordingly, toothed whales reserve their limited resource of air for pneumatic sound production by sequestering air into the thick-walled tracheal and cranial spaces and distensible nasal sacs as the lungs collapse under increasing hydrostatic pressure^15,16^.

Given the small air volume available at depth, echolocating whales are assumed to recycle air from the vestibular sacs back to the nasopharyngeal air space periodically^2,17^ to produce clicks throughout long foraging dives. Although definitive evidence for recycling is absent^17^, it is assumed to occur during occasional pauses in echolocation^18^. If this is correct, deep-diving whales face an apparent tradeoff in how they use air for sound production: investing larger air volumes per click presumably gives greater acoustic output, and thus increased prey detection range, but necessitates frequent recycling and therefore interruption of sensory flow during foraging. The amount of air used in click production has not been measured in any toothed whale but Wahlberg^18^ found that sperm whales paused more frequently when they dove deeper, suggesting that air volume limits continuous click production. Dividing the estimated compressed air volume by the number of clicks between pauses therefore gives an upper bound on the amount of air used per click: some 0.1 litre for a sperm whale at 1500 m depth^18^. Moving this much air through the phonic lips within the approximate 100 μs duration of a click^7^ would require extreme air velocities suggesting that sound production for echolocation may require considerable energy in deep-diving whales, in addition to careful air management to maintain sensory flow.

However, the metabolic cost of echolocation has only been estimated for a relatively shallow diving species, the bottlenose dolphin *Tursiops truncatus* (Montagu, 1821) and with conflicting results. Jensen *et al*.^19^ inferred a cost of 10 mJ/click for wild dolphins based on measured source levels and an assumed production efficiency of 1%. Conversely, a respirometry study on two trained bottlenose dolphins reported that echolocation added about 10% to the metabolic rate of a submerged but still animal^3^, roughly equivalent to 7 J/click. The almost three orders of magnitude difference in these estimates highlights how little we know about toothed whale sound production mechanics. Moreover, animals in both studies had uncompressed lungs and abundant air available for clicking, making the results of questionable relevance to deep-diving echolocators.

Studying sound production at depth in large free-swimming predators is inherently difficult requiring the development of innovative biologging techniques. Here we use a novel non-invasive biologging method that can track nasal air movements related to sound production in wild toothed whales throughout deep dives. Applying the method to short-finned pilot whales, *Globicephala macrorhynchus* (Gray, 1846), we show that their powerful echolocation clicks are produced with extremely small air volumes. Despite this low consumption, air recycling occurs every hundred or so clicks during brief pauses in echolocation. Thus, whales must continually manage air to ensure uninterrupted sensory information flow during prey approaches and capture attempts. The frugal use of recycled air suggests that sound production for echolocation in deep dives is metabolically inexpensive, freeing toothed whales to exploit a wide range of deep-water niches.

## Results

### Dataset

A total of 102 deep foraging dives with a median maximum depth of 685 m were analysed from 23 animals (Supplementary Table 1). Excluding clicks made at less than 200 m depth and clicks occurring after buzzes or communication sounds, these dives contained some 36700 descent clicks in 778 sequences (i.e., separated by a pause). The median number of clicks in these sequences was 36 (IQR: 14–58, max 151). For resonance analysis, these sequences were truncated to between 10 and 129 clicks (median 32) for which a linear model between click sequential number and predicted volume gave a close fit. This reduced the number of analysed clicks to 27404.

### Resonance frequency dynamics

**Figure 1:**
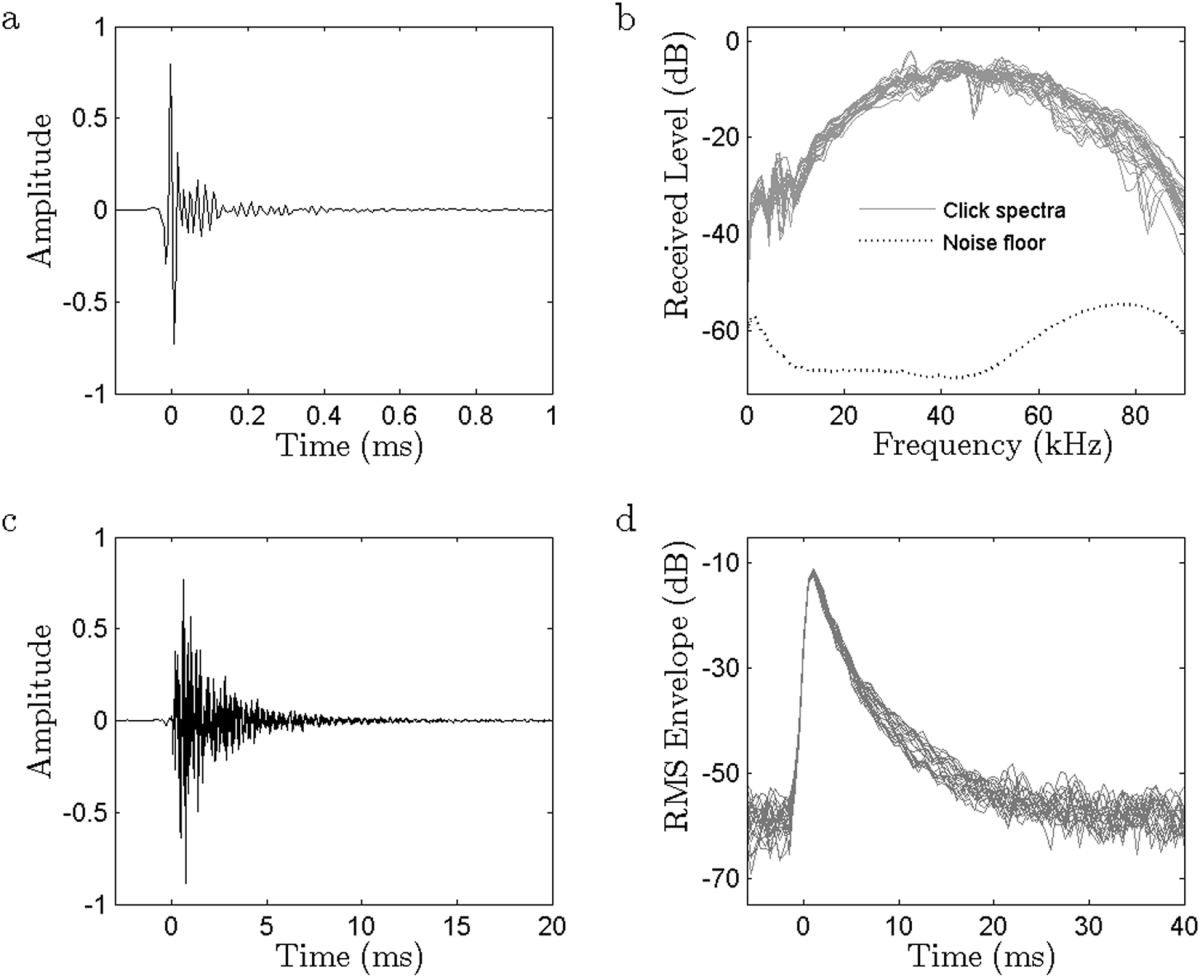
Characteristics of short-finned pilot whale clicks recorded in the far-field and by dorsally-attached tags. (**a**) Waveform of a click recorded in front of a pilot whale and close to the acoustic axis at 530 m depth. (**b**) Spectra of 30 clicks recorded close to the acoustic axis of a pilot whale (256 pt FFT). The dashed line indicates the combined ambient and system noise floor. (**c**) Waveform of a click recorded by a dorsally-attached tag (note the longer time scale compared to panel a). (**d**) The RMS amplitude (1 ms averages) in dB of 20 pilot whale regular clicks recorded by a dorsally-attached tag. All clicks were recorded with 192 kHz sampling rate. Decibels are with respect to an arbitrary reference.

**Figure 2:**
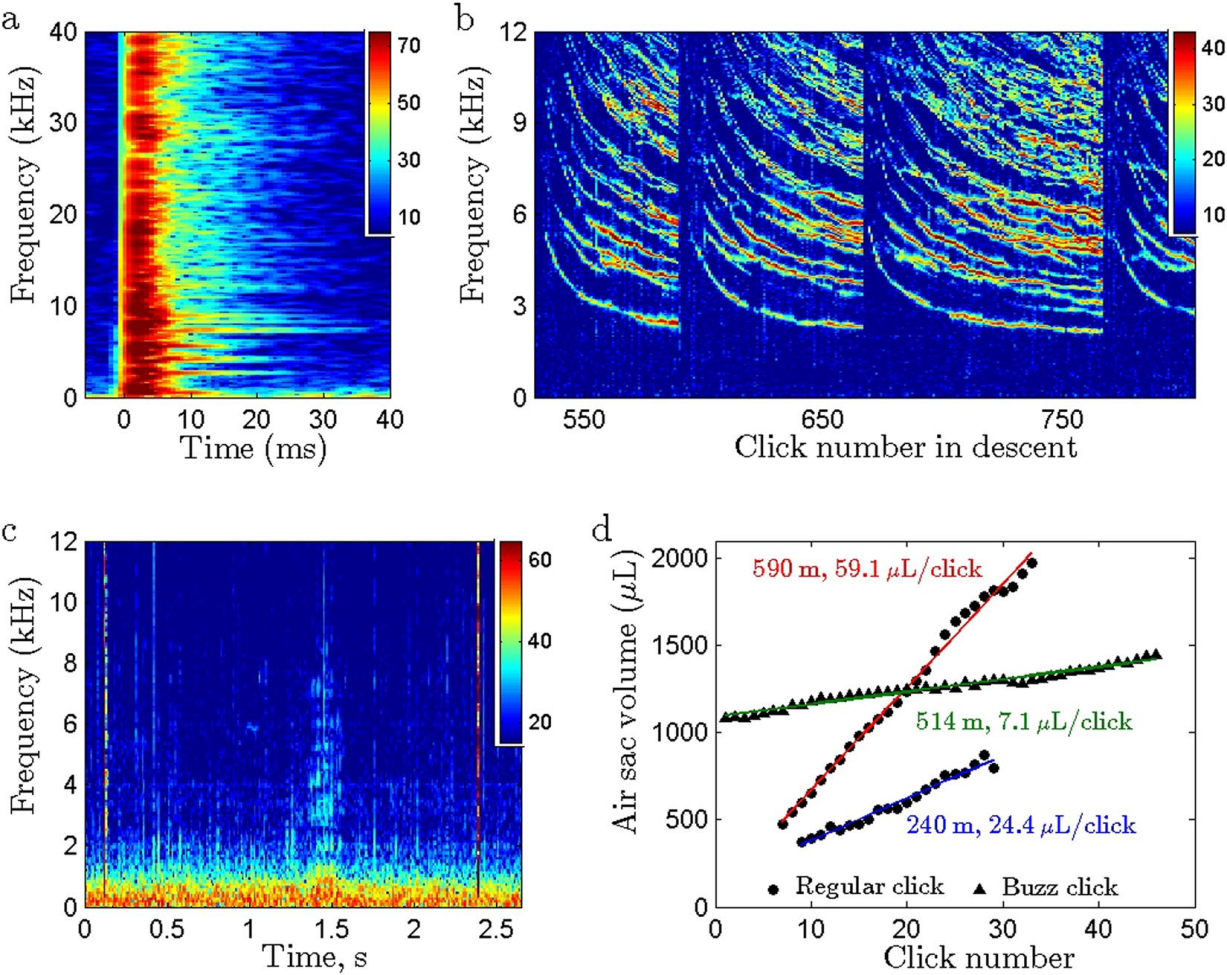
Resonance dynamics of pilot whale clicks recorded by dorsally-attached tags. (**a**) The slow decay of on-animal recorded clicks is due to discrete resonances evident in the spectrogram as narrow horizontal bars following the broadband click (2048 pt FFT, Hamming window, 50% overlap, 192 kHz sampling rate, 500 m depth). Colour in all spectra plots shows the signal-to-noise ratio in dB. (**b**) Stack plot of SNR spectra (47 Hz resolution) for successive clicks during a deep dive descent showing consistent dynamics in resonance frequency: the lowest resonance frequency reduces gradually during each click sequence but makes a step increase after each of the 3 pauses shown. (**c**) A low frequency noise transient is often audible during pauses in clicking (here at 1.5 s), presumably due to air movement (2048 pt FFT, 192 kHz sampling rate, 550 m depth). (**d**) Predicted air space volume for regular click sequences at 240 m (blue regression line, R^2^ = 0.97) and 590 m depth (red regression line, R^2^ = 0.99), and for buzz clicks at 514 m depth (green regression line, R^2^ = 0.97). Air volume per click (the slope of the regression lines) increases with depth and reduces sharply in the lower amplitude buzz clicks.

Clicks recorded by a detached tag in front of a pilot whale that appeared to be actively scanning the tag (Fig. 1a,b) were short transients (median 97% energy duration of 0.14 ms), broadly similar to biosonar signals from other delphinids^20^. The clicks had smooth broadband spectra with a -10 dB range of 20 to 75 kHz although the anti-alias filter in the tag makes this upper limit conservative. In comparison, the clicks recorded by tags attached to whales (Fig. 1c,d) had uniformly slow decay (median 97% energy duration of 4.0 ms; -40 dB envelope duration of 11.7 ms in a subset of 21 clicks from one animal) and the spectra of these clicks showed clear discrete resonance frequencies (Fig. 2a). The decay rate of the lowest resonant component was approx. 0.9 dB/cycle in clicks occurring just before pauses, consistent with a Q of about 30. The frequency of the lowest resonant component, and many of the higher resonances, decreased consistently throughout each click sequence (Fig. 2b): the lowest component started at about 6 kHz and reduced to around 2.5 kHz after 50 clicks. This pattern is consistent with an air space that increases in size with successive clicks. Conversely, a step increase in all resonance frequencies, indicating a sudden reduction in air space size, coincided with almost all pauses. A low frequency noise transient was also consistently audible in some tag recordings during pauses (Fig. 2c), likely related to air movement.

### Air volumes during regular clicking and buzzing

Air volumes predicted using Eq. (3) were closely linear with click number (Fig. 2d), as expected if air volume per click is constant within each sequence. However, the slope of this relation changed with depth. At 500 m depth the average air volume per click, combining α and β in Eq. (5), was 52 μL (46–57 95% CI). This increased/decreased by 10.5 μL (9.9–11.0 95% CI) for each 100 m deeper/shallower than 500 m (Supplementary Table 2). The volume of the vestibular sacs after recycling (i.e., at the start of each sequence) varied widely with a mean of around 700 μL. High marginal and conditional R^2^ of 0.71 and 0.95, respectively, indicate a strong model fit while random effects alone explained little of the data (R_(null)_^2^ = 0.16). Predicted variances were greater for changes in air use within each whale (*σ*_*ε*_^2^) than between whales (*σ*_*k*_^2^) and click sequences (*σ*_*s*_^2^, Supplementary Table 2).

Only one sequence of 46 buzz clicks (mean ICI: 0.02 s) produced at 514 m depth was suitable for quantifying resonances. The apparent output level^21^ of these buzz clicks was on average 16 dB lower than for regular clicks and the air usage per click was 7.1 μL (Fig. 2d).

## Discussion

The capability to detect prey at long ranges using echolocation has been central to the evolutionary success of toothed whales in deep water niches^1^, but has necessitated the development of strategies to produce sound throughout deep dives with a very limited resource of air^10^. Understanding these strategies would shed light on the cost of this active sensory system and the constraints inherited from terrestrial ancestors^22^. However, methods for studying pneumatic sound production in whales under extreme pressures have been absent. Here we show that air use during deep echolocation can be inferred using *in situ* acoustic resonance spectroscopy, the sonic analogue of fluorescence spectroscopy^23^. This non-invasive technique exploits a by-product of click production and requires only the placement of a sound recording tag near the head of an animal. The method can therefore be applied to free-swimming toothed whales performing natural deep foraging dives.

Clicks recorded by tags on echolocating pilot whales have distinctive slow-decaying narrow-band resonances which must be generated by air sacs: reverberations in the skull and soft tissues would decay much more rapidly because of the relatively similar acoustic impedances of these tissues^24^. Although several cranial air spaces could contribute to the resonances, attenuation from the intervening skull limits the detectability of most of these by a dorsally-located tag. Our finding that the lowest resonance frequency reduces with successive clicks indicates that the resonances come primarily from an air space that is receiving air, and so increasing in volume, with each click. This is consistent with the function of the vestibular air sacs^17^ while the dorsal location of these sacs near the vertex of the skull ensures a sound path for resonances to reach the tag. Click-by-click variation in the lowest resonance frequency recorded by the tag therefore provides an opportunity to measure the volume of air delivered to the vestibular sacs with each click as a function of depth.

The resonance frequency dynamics indicate that at 500 m depth about 50 μL of air are used to produce each click, with the low variance of this value indicating relatively uniform air use across individuals. This volume increases with depth leading to twice as much air being used per click at 1000 m. Nonetheless, the volumes are extremely small, supporting the hypothesis that echolocation clicks can be produced pneumatically throughout deep foraging dives from a strictly limited air supply^9^. But can such small air volumes deliver enough energy to produce intense echolocation clicks? Although the source parameters of short-finned pilot whale clicks are not known, extreme values for a similarly-sized odontocete, the false killer whale *Pseudorca crassidens* (Owen, 1846), are an on-axis source level of 208 dB re 1 μPa RMS at 1 m, click duration of 50 μs, and directivity index of 29 dB^25^. With these parameters, each click contains an acoustic energy of about 0.3 mJ (see Jensen *et al*.^19^ for a similar computation). In comparison, the work done by a volume of compressed air expanding adiabatically to ambient pressure, *p*_*A*_, from an initial pressure of *p*_*A*_ + *δ*_*p*_ is: $W\approx 0.7{v}_{f}\cdot {\delta }_{p}$, where *v*_*f*_ is the uncompressed volume and *δ*_*p*_ is assumed to be small compared to *p*_*A*_. As energy is lost converting mechanical to acoustical work, *W* must be greater than the acoustical energy of a click. Given a conversion efficiency *ζ*, the pressurization needed to generate a 0.3 mJ pilot whale click with 50 μL (i.e., 50 × 10^−9^ m^3^) of air is:

1 $${\delta }_{p}\approx 0.3\cdot {10}^{-3}/(0.7\cdot 50\cdot {10}^{-9}\cdot \zeta )\approx 8600/\zeta \,Pa$$

Inter-narial pressure during echolocation in bottlenose dolphins, measured by inserting pressure catheters into the blowholes of partially submerged animals, varies between 25 and 80 kPa^2,26^. Using these values in (1) gives a mechanical efficiency of 11–34% which, combined with a typical 25% efficiency for converting metabolic to mechanical energy in muscles, implies an overall efficiency of 3–9%. Vocal efficiency has not directly been measured in toothed whales and numbers vary widely for animals calling in air: from ≪1% for humans^27^ to 0.8–5% for frogs^28^. While pneumatic sound production could conceivably be more efficient in water than in air, higher inter-narial pressures are also readily attained by animals clicking at depth, lowering the efficiency needed to achieve a given acoustic output. For example, at 500 m depth a 5% contraction of the nasopharyngeal air space would yield an inter-narial pressure of 250 kPa, sufficient to drive the presumed acoustic output with an overall efficiency of 0.9% and a metabolic cost per click of about 40 mJ. Thus the small air volumes found here to be involved in deep echolocation are able to store enough mechanical energy to produce intense clicks with only moderate pressurization and a low conversion efficiency.

Using a rough estimate of 1% efficiency, the 1000 or so regular (i.e., non-buzz) clicks produced by pilot whales during a deep dive have a total metabolic cost of some 40 J per dive. In comparison, the mechanical work done in compressing air isothermally in the lungs when diving from the surface to depth is ${p}_{0}{v}_{0}ln({p}_{1}/{p}_{0})$, where p_0_ and p_1_ are the surface and final pressures and v_0_ is the surface volume, i.e., about 37 kJ for a 900 kg pilot whale with a 90 litre lung volume^29^ diving to 600 m. Although this is somewhat offset by the terminal glide in the ascent powered by the expanding lungs, the high energy and therefore low efficiency stroking needed to depart the first few metres from the surface^11^ is unlikely to be recouped. The net metabolic cost of lung compression is therefore likely to be many 10’s of kJ per dive and could represent a substantial component of the total energetic cost of diving. If air was not needed for sound production this expense could be avoided by diving on exhalation as done by some pinnipeds^30^ making the whales more closely neutrally buoyant. Thus, sound production represents a very small part of the energy budget of a foraging dive, and the work done against lung buoyancy, needed to provide the air reservoir for sound production, is likely the dominant cost of echolocation.

Despite the small air volume expended per click, pilot whales need to recycle air frequently at depth. Periodic upward steps in the resonance frequencies (Fig. 2b) indicate that air is recycled from the vestibular sacs back into the nasopharyngeal air space during pauses. The vestibular sacs are not completely emptied as it would be inefficient to do so: our model suggests an average of about 700 μL (Supplementary Table 2) is left in the sacs. Recycling pauses are accompanied by a puff sound in some tag recordings presumably generated by air passage through the phonic lips and similar sounds have been noted during echolocation pauses in other deep-diving toothed whale species tagged with the same devices. In our pilot whale dataset, 99% of pauses longer than 1 s were associated with recycling, and we therefore propose that such pauses in echolocation are generally indicative of air recycling in toothed whales.

The tagged pilot whales produced no more than 150 regular clicks between recycling pauses giving a vestibular sac volume at maximum inflation of some 9 cm^3^ at 500 m depth. This may be well less than the maximum capacity of these sacs as the variability in click sequence length suggests that pilot whales elect when to recycle based on their foraging state. To verify this, the left vestibular sac of a recently expired sub-adult short-finned pilot whale stranded in the Canary Islands was measured from a cast made by injecting silicone through the nares following Nakamura *et al*.^31^. The volume of the excised cast was estimated at 45 cm^3^, substantially larger than the peak air volumes predicted in this study. Elective recycling pauses generally occurred during regular clicking rather than after buzzes and so may be timed to ensure readiness for extended clicking during chases after energetic prey^32^.

An unexpected prediction from our model is that air volume per click increases with depth at a rate of some 10 µL per 100 m. This result was consistent across the 23 individuals in the study suggesting that it is a fundamental property of the sound production mechanism. Although increasing volume could imply that stronger clicks are produced at depth this seems unlikely as higher sound levels would be more useful at the start of descents when the distance to prey is greatest. The effect therefore seems more likely to result from a mechanical constraint, for example, slower phonic lip closure as the density of compressed air increases. In any case, a consequence is that recycling may need to be performed more frequently as depth increases, as has been observed in sperm whales^18^. If a similar constraint applies to beaked whales that can reach depths in excess of 3 km^33,34^, pauses may be required so frequently that continuous tracking of prey is compromised. However, resonance analysis of buzz clicks hint at another way in which animals can manage air usage during long sequences of continuous clicking. All studied toothed whales make rapid click sequences or buzzes during prey capture attempts when foraging with echolocation^35^. These contain 100’s of clicks with intensity some 15–20 dB less than for regular search clicks^21^. Although resonances are difficult to measure during rapid buzz clicking, we found that air volume per click dropped by nearly an order of magnitude at the start of the one analysable buzz in our dataset, consistent with the reduction in output power. This suggests that echolocators control click intensity and air usage in unison, enabling long click sequences without recycling when prey are close.

We have demonstrated that on-animal recordings of echolocation clicks provide an unanticipated source of data that would otherwise be extremely difficult to obtain on how air in nasal spaces is used during deep dives. These data reveal that pilot whales, and likely other toothed whales, use two tactics to produce long sequences of echolocation clicks during foraging dives: small air volumes are used to produce each click, and the collected air is periodically recycled to allow nearly continuous sound production at low metabolic cost. Precise management of air may be aided by adjusting click intensity and by choosing to recycle air when sensory input from echolocation is less critical. These tactics have enabled toothed whales to harness an extraordinarily powerful sensory system despite the seeming disadvantages of air-propelled sound production for an aquatic animal at depth.

## Materials and Methods

### Tagging

Tags were deployed on short-finned pilot whales off the southwest coast of Tenerife (Canary Islands, Spain) in 2003, 2004, 2006 & 2008. Tagging was conducted under permit from the Canary Islands Government to N.A.S with an animal experimental protocol approved by the Woods Hole Oceanographic Institution Animal Care and Use Committee (prior affiliation of M.J). All methods were performed in accordance with the relevant guidelines and regulations.

Pilot whales were approached by converging slowly on their course from a 12 m boat, and a sound and movement recording tag (DTAG, version 2) was placed on the dorsal surface using a 5 m long hand-held carbon fibre pole. Tags were attached to the whales with 4 suction cups which vented after a programmed time to release the tag for recovery. DTAGs are archival devices equipped with a pressure sensor, triaxial accelerometers and magnetometers all sampled at 50 Hz^36^. They also record sound from one (2003) or two hydrophones at 96 or 192 kHz with 16-bit resolution. Although pilot whale clicks extend well beyond the 48 kHz frequency range of a tag sampling at 96 kHz, the resonant phenomena of interest here have frequencies <10 kHz and so are well within the bandwidth of the tags.

### Data analysis

Data from whales performing two or more deep (>500 m) dives were analysed using custom scripts in MATLAB 8.5 (MathWorks). The emission times of regular echolocation clicks^32^ produced by the tagged animal during deep dives were determined using a supervised click detector. Only clicks produced in the descent and below 200 m depth were analysed since social calls are frequent in ascents and at shallow depths^10,37^. Dives containing less than 200 clicks produced during the descent were excluded from analysis.

Working on the hypothesis that pauses in clicking are associated with air recycling, we defined pauses as occurring whenever the inter-click interval (ICI) was greater than two times the mean ICI of the two previous and two following clicks. Subsequent analyses of click waveforms indicated that recycling occurred in all but a small number (1.4%) of these detected pauses. Pauses found not to be associated with recycling were thereafter eliminated. Regular clicks were distinguished from buzzes (fast click series associated with prey capture attempts) and other rapid click sequences using an ICI threshold of 0.2 s^32^. Since air use to produce tonal signals and rapid click sequences may differ from that of regular echolocation clicks^4^, any regular clicks after buzzes or communication signals such as rasps and squeaks^37^ until the next pause (i.e., putative recycling event) were excluded.

Clicks recorded by tags attached to animals generally have very different characteristics than clicks recorded in the far-field due to the location of the tag close to, but behind, the sound source^38^. The on-axis characteristics of short-finned pilot whale clicks have not been described and to address this, we used data from a tag (192 kHz sampling rate) which detached from a whale at 530 m depth during a foraging dive. This tag separated from the animal due to loss of vacuum in the suction cups and proceeded to float away. The tag recording shortly after detachment contained a series of high level clicks with rising and falling amplitude consistent with echolocation scans^39^ suggesting that the pilot whale was inspecting the tag. 31 clicks that appeared to be recorded close to the acoustic axis of the whale (judging by their higher received level compared to preceding and subsequent clicks) were chosen from this encounter to approximately characterise the far-field waveform and spectra. Spectra of clicks were computed using a 256 point FFT (rectangular window) while the ambient noise spectrum was estimated from a 1.3 s sample from the same recording using a 256 point FFT (50% overlap, Hann window). Click length was estimated from the 97% energy duration.

Compared to far-field clicks, clicks recorded while tags were attached to pilot whales had greatly extended durations. Inspection of spectrograms indicated that this was due to a set of slow-decaying narrow-band components indicative of a resonant phenomenon. To examine the frequency and potential source of this resonance, we quantified the spectrum of slow-decaying components following each click by computing a 2048 (96 kHz sampling rate) or 4096 (192 kHz sampling rate) point FFT starting 7 ms after the start of the click. This offset was chosen to avoid the click itself as well as fast-decaying reverberation e.g., from cranial structures. To partly compensate for the exponential decay of the signal over the 21 ms (i.e., 2048 samples at 96 kHz) analysis window, a non-symmetric half Bartlett window was used. Ambient noise in the 50 ms prior to each click was estimated by averaging the spectral power (Hann window, 50% overlap) in sequential 512 (at 96 kHz) or 1024 (at 192 kHz) point FFTs. Noise spectra were then interpolated to 2048 or 4096 points and used to normalize the click FFTs resulting in a signal-to-noise ratio (SNR) spectrum with 47 Hz resolution. For tags with two hydrophone channels, the spectral powers for each channel were averaged. To visualise how resonance frequencies change during click sequences, successive SNR spectra during a dive were assembled in stack plots. A supervised Kalman filter peak tracker was used to identify the lowest resonance frequency in these images. The FFT bin with the highest level in a window of +/− 4 FFT bins (approx. 188 Hz) around each track point was then located, and cubic interpolation was performed on this bin and its two neighbours to estimate the precise resonance frequency for each click.

To study click resonances during buzzes, the same analysis was used but with a shorter 1024 (96 kHz) point FFT starting 3 ms after the start of each click. This enabled analysis of clicks with ICI as low as 14 ms. The ICI in pilot whale buzzes typically decreases rapidly from a nominal starting value of 0.2 s (Aguilar de Soto *et al*. 2008) to <5 ms. Resonance could only therefore be examined in buzzes with unusually large ICI of which only a single example with acceptable SNR was found in the dataset.

We hypothesized that the resonances in pilot whale clicks are generated by one or more of the nasal air sacs when excited by broadband clicks. Similar acoustic resonances occur in gas-filled swim bladders of fish when insonified by echosounder pulses^40^. Assuming that the air volume is roughly spherical, the fundamental resonance frequency (*f*_0_) of a swim bladder can be approximated by^41^:

2 $${f}_{0}=\frac{1}{2\pi r}\cdot \sqrt{\frac{3{\gamma }_{a}{P}_{w}}{\rho }}$$

where *γ*_α_ is the ratio of specific heats of air (i.e., 1.4) and *r* is the radius of the sphere (m); *P*_*w*_ is the ambient water pressure (Pa) and *ρ* is the density (kg/m^3^) of the tissue surrounding the swim bladder. In applying this formula to pilot whale nasal air sacs, we took a tissue density of 1087 kg/m^3^ ^42^, and assumed that this and *γ*_α_ vary negligibly with depth. Resonance frequency is therefore a function of effective air sac radius (i.e., the cube-root of volume) and pressure, such that the air sac volume can be predicted from 1/*f*_0_^3^ as:

3 $$\hat{V}=\frac{1}{{f}_{0}^{3}}\cdot {P}_{w}^{1.5}\cdot \frac{4\pi }{3}\cdot {(\frac{3{\gamma }_{a}}{4{\pi }^{2}\rho })}^{1.5}$$

### Statistical modeling

If our hypothesis is correct, the predicted air sac volume should change with successive clicks as air used in the production of each click is moved in or out of the sac. We assumed that each click within a click sequence involves movement of a roughly constant (but potentially depth dependent) volume of air, such that air sac volume is proportional to click number, counting from the last presumed recycling event. A simple parametric model for air sac volume can be formulated by assuming that air sacs are approximately spherical so that the relationship between the inverse cube of resonant frequency and volume (Eq. (3)) is close to linear. As click sequences are typically <30 seconds long, changes in air volume due to the change in depth during a sequence can be neglected. Under these assumptions, a linear model for air sac volume in each click sequence is:

4 $$\widehat{{V}_{i}}={V}_{0}+\alpha \cdot i+{\varepsilon }_{i}$$

where *i* is the click number in the sequence with 1 corresponding to the first click after recycling; ${\hat{V}}_{i}\,$is the predicted air sac volume after click *i* (calculated with Eq. (3)); *V*_0_ is the intercept (i.e., the volume of the air sacs at the start of the sequence after recycling); α is the volume of air added to the sacs (if receiving air), or subtracted from the sacs (if the source of air), with each click; and *ε*_*t*_ is the volume prediction error for each click.

The assumption of a spherical air volume is likely only applicable when the air sacs are small and unconstrained by surrounding tissue (elongate swim bladders have a higher resonance frequency for the same volume^43^). We therefore expect a linear relationship between click number and air volume for only a subset of clicks within each click sequence when the air space is small: this relationship will change when the air space is larger and therefore more constrained in shape. Click resonances in this study are consistent with an air space that receives air during clicking, and the linear subset should therefore occur at the start of each click sequence when the air space is small. To identify clicks from each sequence to include in the linear model, we compared the R^2^ of 1st and 2nd order model fits between click number and $\hat{V}$ for decreasing subsets of clicks within each sequence. The largest number of clicks for which the R^2^ of the two models were within 0.01 was chosen. A few click sequences with less than 10 clicks meeting this criterion and with R^2^ less than 0.9 were rejected.

A linear mixed-effects model was then formulated to analyse predicted air sac volumes from multiple animals and click sequences. To allow for variable amounts of air in the air sacs following each recycling, within-animal and within-sequence correlations in air use were treated with random intercepts, with sequences nested within each animal. The resulting model is:

5 $${\hat{V}}_{ijk}=({V}_{0}+{u}_{j}+{u}_{k})+\alpha \cdot i+\beta \cdot i\cdot {d}_{ijk}+{\varepsilon }_{ijk}$$

where *j* is the click sequence number for each animal; *k* is the individual pilot whale; ${\hat{V}}_{ijk}$ is the predicted air sac volume for click *i*, in sequence *j*, produced by whale *k*, calculated using (3); *u*_*j*_ and *u*_*k*_ are the random effects for sequence and animal respectively; α is the volume change of the sacs with each click; *d*_*ijk*_ is the depth at which each click is produced; and *ε*_*ijk*_ are the residuals. An interaction term, β, is included for depth and click number to allow for depth-dependent click volumes. The average volume of air added to the sacs with each click is therefore α + β *d*_*ijk*_.

To ameliorate potential serial correlation in the repeated measures we used an AR(1) correlation structure in solving this LMM. Goodness of fit was evaluated using the marginal (R_(m)_^2^), and conditional R^2^ (R_(c)_^2^)^44^. These describe the proportion of the variance in the response variable that is explained by the fixed variables only, and the entire model (fixed plus random effects), respectively. The R^2^ for the null model (i.e., the model containing the same random structure but no fixed variables) was also computed to describe the proportion of variance that is explained by the random variables only. Statistical analyses were made with RStudio and package nlme.

## Supplementary information

Supplementary data

Dataset

## Data Availability

The dataset generated and analysed during the current study is included in this published article (and its Supplementary Information Files).
