# Supplementary material for: Deep-diving pilot whales make cheap, but powerful, echolocation clicks with 50 µL of air
Source: Sci Rep. 2019 Oct 31;9:15720. doi: 10.1038/s41598-019-51619-6 (PMC6823382; doi:10.1038/s41598-019-51619-6)
Supplement: Supplementary file 1 — Supplementary data [file 41598_2019_51619_MOESM1_ESM.doc]

**Supplementary material**

**Deep diving pilot whales make cheap, but powerful, echolocation clicks with 50 µL of air**

Ilias Foskolos, Natacha Aguilar de Soto, Peter Teglberg Madsen, Mark Johnson^*^

*Corresponding author email: markjohnson@st-andrews.ac.uk

**Supplementary Table 1:** Data used in resonance analysis. Animal ID comprises year, Julian day and a letter denoting encounter of day. #dives is the number of descents of dives deeper than 500 m for which resonances were measurable. #click seqs is the number of sequences of clicks analysed from these dives. Each sequence starts after a pause and ends either with a pause, a buzz or a non-click vocalization. #clicks is the total number of clicks for which resonance frequency measurements were made for each animal.

| Date | ID | #dives | #click seqs | #clicks | depth range (m) |
| --- | --- | --- | --- | --- | --- |
| 02/11/2003 | 03_306a | 3 | 10 | 311 | 405-697 |
| 02/11/2003 | 03_306b | 11 | 118 | 3677 | 202-726 |
| 02/11/2003 | 03_306d | 3 | 13 | 224 | 202-575 |
| 03/11/2003 | 03_307a | 4 | 29 | 1271 | 207-689 |
| 03/11/2003 | 03_307b | 4 | 11 | 301 | 230-657 |
| 04/11/2003 | 03_308a | 2 | 25 | 911 | 295-802 |
| 04/11/2003 | 03_308b | 5 | 38 | 1248 | 361-709 |
| 04/11/2003 | 03_308c | 10 | 94 | 4173 | 200-645 |
| 05/11/2003 | 03_309c | 2 | 10 | 276 | 212-692 |
| 22/10/2004 | 04_296a | 9 | 36 | 1357 | 268-664 |
| 22/10/2004 | 04_296b | 3 | 21 | 686 | 295-620 |
| 22/10/2004 | 04_296d | 2 | 5 | 167 | 212-421 |
| 23/10/2004 | 04_297i | 2 | 27 | 866 | 238-628 |
| 25/10/2004 | 04_299c | 3 | 18 | 590 | 202-535 |
| 22/03/2006 | 06_081e | 7 | 46 | 1688 | 208-593 |
| 26/03/2006 | 06_085h | 2 | 8 | 187 | 438-638 |
| 17/04/2008 | 08_108d | 7 | 56 | 1592 | 280-646 |
| 19/04/2008 | 08_110b | 5 | 45 | 1643 | 234-663 |
| 19/04/2008 | 08_110c | 2 | 9 | 219 | 526-636 |
| 19/04/2008 | 08_110d | 8 | 99 | 3999 | 204-687 |
| 21/04/2008 | 08_112e | 3 | 14 | 338 | 254-514 |
| 22/04/2008 | 08_113c | 3 | 19 | 601 | 475-720 |
| 22/04/2008 | 08_113e | 2 | 27 | 1079 | 271-627 |

**Supplementary Table 2:** Summary of the fixed and random effects for the full linear mixed model (equation (5)), and the goodness of fit. Est: estimate; CI: confidence intervals; *σ_ε_^2^*, *σ_k_^2^* and *σ_s_^2^*: the variances within and between animals and between click sequences respectively; atm: 1 atmosphere (i.e., 10 m). Note that the small value and lack of significance of *α* in the model fit means that air usage per click is dominated by *β,* the coefficient of the interaction term with depth. Thus, the mean air usage at 500 m depth is 52 μL (i.e., 1.05 x 51 atm) and this increases by 10.5 μL for each additional 100 m depth.

|  | **Fixed effects** | | | |
| --- | --- | --- | --- | --- |
| **Parameters** | **Units** | **Est** | **95% CI** | **p-value** |
| *V_0_* | μL | 677 | [427, 927] | 0 |
| *α* | μL/click | -1.55 | [-4.31, 1.20] | 0.27 |
| *β* | μL/click∙atm | 1.05 | [0.99, 1.10] | 0 |
| *σ_ε_^2^* | 91∙10^4^ | | | |
|  | **Random effects** | | | |
|  | **Est** | | **95% CI** | |
| *σ_k_^2^* | 36∙10^5^ | | [19∙10^5^, 66∙10^5^] | |
| *σ_s_^2^* | 12∙10^5^ | | [10∙10^5^, 14∙10^5^] | |
|  | **Goodness of fit** | | | |
|  | R_(m)_^2^ | 0.71 | R_(c)_^2^ | 0.95 |

**Supplementary Table 3: Summary of the variables that were used during modelling for the estimation of air volume per echolocation click.**

| **Variables** | **Description** |
| --- | --- |
| *an_id* | unique id number for each whale |
| *seq_id* | sequential number for click sequences produced by a whale |
| *cl_n* | sequential click number for clicks within click sequences (measuring from the last pause in clicking) (1^st^ independent variable) |
| *dep* | depth of click production |
| *dep_10* | depth of click production divided by 10 |
| *dep_10_cl_n* | interaction term (product) between *dep_10* and *cl_n* (2^nd^ independent variable) |
| *l_res_khz* | the lowest resonant frequency in kHz |
| *vol_ul* | the calculated volume of the vestibular air sacs in μL (dependent variable) |

**
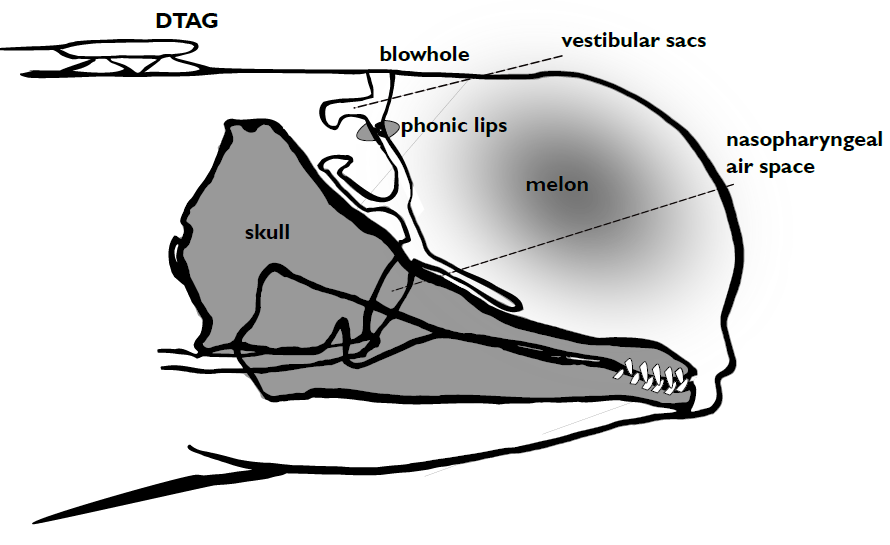
**

**Supplementary Figure 1:** Schematic of a short-finned pilot whale head. Echolocation clicks are produced by passing compressed air from the nasopharyngeal air space through the bony nares to the vestibular sacs via the phonic lips. Resonances in pilot whale clicks are generated by one or more of the nasal air sacs when excited by broadband clicks and are recorded by hydrophones in the dorsally-attached DTAG (Copyright: Catherine Williams).
